# Transcriptome and Gut Microbiota Profiling Analysis of ANIT-Induced Cholestasis and the Effects of Da-Huang-Xiao-Shi Decoction Intervention

**DOI:** 10.1128/spectrum.03242-22

**Published:** 2022-11-21

**Authors:** Wang Wang, Shujun Jiang, Chengcheng Xu, Lili Tang, Yan Liang, Yang Zhao, Guoxue Zhu

**Affiliations:** a Department of Neurology, Nanjing Hospital of Chinese Medicine Affiliated to Nanjing University of Chinese Medicine, Nanjing, Jiangsu, China; b School of Medicine & Holistic Integrative Medicine, Nanjing University of Chinese Medicine, Nanjing, Jiangsu, China; c Chinese Medicine Modernization and Big Data Research Center, Nanjing Hospital of Chinese Medicine Affiliated to Nanjing University of Chinese Medicine, Nanjing, Jiangsu, China; Nanjing Institute of Geography and Limnology, Chinese Academy of Sciences

**Keywords:** cholestasis, Da-Huang-Xiao-Shi decoction, transcriptome, microbiome, Pearson correlation analysis

## Abstract

Cholestasis is characterized by bile acid (BA) circulation disorders, which is usually related to damage of hepatocyte barrier function. Currently, patients with cholestasis face several obstacles in seeking diagnosis and therapy. Da-Huang-Xiao-Shi decoction (DHXSD) is an ancient classic formula that has been used clinically for cholestasis treatment. Nevertheless, the underlying biological activities and therapeutic mechanisms remain unclear. In this study, an alpha-naphthylisothiocyanate (ANIT)-induced cholestasis rat model was established to examine the anticholestatic effects of DHXSD using histopathological and molecular analyses. Transcriptomic analysis combined with 16S rRNA gene sequencing analysis was systematically applied to study the mechanism of action of DHXSD. Simultaneously, the effect of DHXSD on gut microbiota, short-chain fatty acids (SCFAs), and intestinal barrier function were evaluated based on the ANIT-induced cholestasis model in rats. The results showed that DHXSD effectively attenuated ANIT-induced cholestasis by reducing liver function indicators (alanine transaminase [ALT], *P* < 0.05; alkaline phosphatase [ALP], *P* < 0.05; total bile acid [TBA], *P* < 0.01; γ-glutamyl transpeptidase [GGT], *P* < 0.001) and levels of hepatotoxicity-related enzymes (*P* < 0.05), thus improving the recovery of histopathological injuries, and regulating levels of inflammatory cytokines (*P* < 0.05). In addition, 16S rRNA gene sequencing analysis combined with intestinal barrier function analysis revealed that the DHXSD significantly ameliorated ANIT-induced gut microbiota dysbiosis. Significantly altered genes in the model and treatment groups were screened using transcriptomic analysis. Sixty-eight genes and four microbial genera were simultaneously altered with opposing trends in variation after ANIT and DHXSD treatments. We built a framework for predicting targets and host-microbe interaction mechanisms, as well as identifying alternative treatment for cholestasis, which should be validated further for clinical application. In conclusion, DHXSD appears to be a promising agent for protection against liver injury.

**IMPORTANCE** Cholestasis is a serious manifestation of liver diseases resulting in liver injury, fibrosis, and liver failure with limited therapies. To date, only ursodeoxycholic acid (UDCA) has been approved by the U.S. Food and Drug Administration for the treatment of cholestasis. However, approximately one-third of patients with cholestasis are unresponsive to UDCA. Therefore, it is urgent to search for appropriate therapeutic agents for restoring stoppage status of the bile components to treat cholestasis. In this study, we investigated how the microbiome and transcriptome data sets correlated with each other to clarify the role of microbiome alterations in host metabolism. In combination, this research offers potential molecular biomarkers that should be validated for more accurate diagnosis of cholestasis and the clinical utilisation of gut microbiota as a target for treatment.

## INTRODUCTION

Cholestasis is mainly characterized by inhibition of bile flow resulting from a variety of mechanisms, such as inhibition of the bile salt export pump (BSEP), ductular obstruction, and intracellular calcium homeostasis alteration (1, 2), and may affect approximately 10 to 20% of the overall population. Currently, the main clinical treatment methods for cholestasis include ursodeoxycholic acid (UDCA) and S-adenosyl-l-methionine (SAMe) administration (3). The current absence of therapeutic drugs for cholestasis are due to a lack of understanding of the complex molecular mechanisms of the disease (4). Cholestasis is likely to result in acute liver toxicity and jaundice and aggravated outcomes such as hepatitis, hepatic fibrosis, cirrhosis, and even liver cancer without effective pharmacotherapy (5). Consequently, the discovery and development of new drugs for cholestasis therapy is needed. Alpha-naphthylisothiocyanate (ANIT) is an indirect hepatotoxic agent which is regarded as a well-accepted theoretical model to study cholestasis. Although mechanisms of ANIT-induced cholestasis have been proposed, it has not been entirely elucidated. ANIT-induced liver injury is mediated an ANIT-glutathione (ANIT-GSH) conjugate which decouples upon crossing the canalicular membrane and produces free GSH and ANIT in bile. Furthermore, the excretion of ANIT and GSH into the bile duct damages biliary epithelial cells and induces cholestasis (6, 7).

Traditional Chinese medicine (TCM), a multicomponent and multitarget system, has become an important strategy for the clinical treatment of a variety of chronic diseases, including liver diseases (8, 9). Da-Huang-Xiao-Shi decoction (DHXSD) was originally described by Jin Gui Yao Lue in *Synopsis of Prescriptions of the Golden Chamber*; it comprises *Rheum officinale* Baill (Rhubarb, DH), *Gardenia jasminoides* Ellis (Fructus Gardeniae, ZZ), *Phellodendron chinense* Schneid (Cortex Phellodendron, HB), and Mirabilitum (a mineral medicine, MX), with a mass ratio of 4:3:4:4. DHXSD is a representative formula for the treatment of damp-heat jaundice in long-term clinical practice. Some compounds and active ingredients of DHXSD have shown superiority in the treatment of cholestasis. For example, anthraquinones protect hepatocytes and cholangiocytes against ANIT-induced damage by promoting farnesoid X receptor (FXR) signaling and anti-inflammatory pathways (10–12). Iridoid glycosides, the main constituent of ZZ (geniposide, for example), can improve ANIT-induced damage by regulating bile transporters (such as BSEP and MRP2), bile acids biosynthesis (CYP7A1, CYP8B1, and CYP27A1), oxidative stress, and the inflammatory response (13–15). Alkaloids (the main active ingredients of HB) have shown anti-inflammatory, hepatoprotective, and antioxidant effects, which is the key to the treatment of cholestasis (16).

The anatomy of the gut is closely related to that of the liver through the portal vein. With the development of the gut-liver axis theory (one of the basic foundations of TCM theory and guiding its clinical application), previous studies have illustrated that the liver and intestinal tract could affect one another based on both physiological and pathological conditions (17–19). Since ancient times, DHXSD has been an important TCM formula utilized to treat intestinal diseases. Therefore, integration appraisement on the usage of DHXSD in treating liver injury based on the perspective of “gut-liver axis” has vital clinical implications. With the development of various omics technologies, their combined application and integrated analysis of omics-based data sets provide new insights into our understanding of the intestine and its role in liver injury development (gut-liver axis theory). Compared with the whole methods, the advantages of transcriptome analysis are obvious, such as the contribution of new strategies for drug discovery. Here, we performed a systematic study on the anticholestasis mechanism of DHXSD by combining gut microbiota and transcriptomic analyses.

## RESULTS

### DHXSD exhibited therapeutic effects on cholestasis.

**FIG 1:**
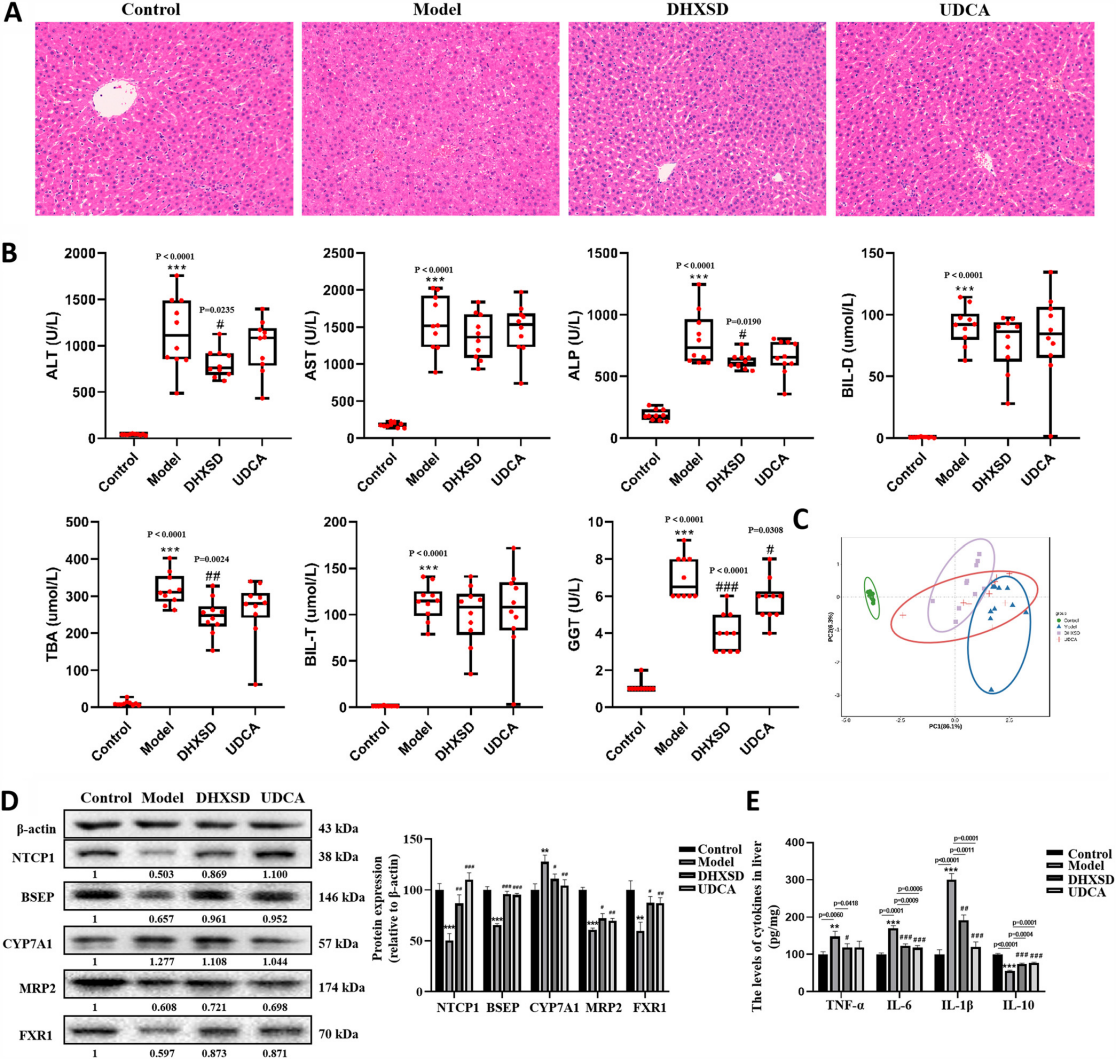
(A) Effects of DHXSD on histological changes (100× magnification) and (B) serum biochemistry. (C) PCA analysis of seven liver function indexes. (D) Effects of DHXSD on hepatic protein expression of uptake and efflux transporters. (E) The levels of inflammatory cytokines TNF-α, IL-1β, IL-6, and IL-10 in liver were detected by ELISA. *, *P* < 0.05; **, *P* < 0.01; ***, and *P* < 0.001 compared with the control group; ^#^, *P* < 0.05; ^##^, *P* < 0.01; and ^###^, *P* < 0.001 compared with the model group.

Histological analysis provided direct evidence of the protective effect of DHXSD against ANIT-induced liver injury. As shown in Fig. 1A, the hepatic tissues of the control group displayed a normal cell structure and were free of abnormal morphological changes. Nevertheless, rats in the ANIT group showed acute infiltration, edema, and hepatic necrosis. A mild degree of bile duct epithelial damage and hepatocyte hydropic degeneration of liver injury was observed in the UDCA and DHXSD groups, which was similar to that in the control group.

As shown in Fig. 1B, compared with rats in the control group, the model group rats that were intoxicated by ANIT showed a remarkable increase in the levels of the sensitive indices of liver damage (ALT and aspartate transaminase [AST]) and crucial indices of cholestasis (ALP, total bilirubin [BIL-T], direct bilirubin [BIL-D], TBA, and GGT). Administration of DHXSD significantly reduced the serum levels of ALT, ALP, TBA, and GGT, which was nearly equal to the results obtained by administering UDCA. Principal-component analysis (PCA) was conducted to comprehensively estimate the influence of the seven liver function indices. As shown in Fig. 1C, the clustering significantly differed between the control, ANIT, DHXSD, and UDCA groups, demonstrating a significant disparity in liver status among these groups. Simultaneously, the distance from the control group to the model group was significantly larger than that from the DHXSD group. In addition, the distance from the control group to the DHXSD group was similar to that from the UDCA group. This indicated that DHXSD had a regulatory effect on comprehensive liver function like that shown by UDCA.

Cholestasis is one of the most common liver diseases and is characterized by BA circulation disorders. To evaluate the influence of cholestasis and DHXSD treatment on BA circulation, the protein levels of uptake and efflux transporters in rats, including NTCP, CYP7A1, FXR, MRP2, and BSEP, were quantified. As shown in Fig. 1D, the protein levels of NTCP, CYP7A1, FXR, MRP2, and BSEP were downregulated in the ANIT-induced model group compared to in the control group, while DHXSD treatment remarkably reversed this trend.

To evaluate the effect of DHXSD treatment on the inflammatory response, concentrations of inflammatory cytokines (TNF-α, IL-6, IL-1β, and IL-10) were measured in the liver. The concentrations of the proinflammatory factors TNF-α, IL-6, and IL-10 (Fig. 1E) in the model group were significantly higher than in the control group. DHXSD treatment significantly reduced ANIT-induced increases in these cytokines. Compared to the control group, the level of the anti-inflammatory factor IL-10 (Fig. 1E) in the ANIT-induced model group was significantly lower. However, after treatment with DHXSD, the concentration of IL-10 showed an opposite trend.

### DHXSD protected the integrity of the colonic intestinal epithelial layer in ANIT-treated cholestatisis.

**FIG 2:**
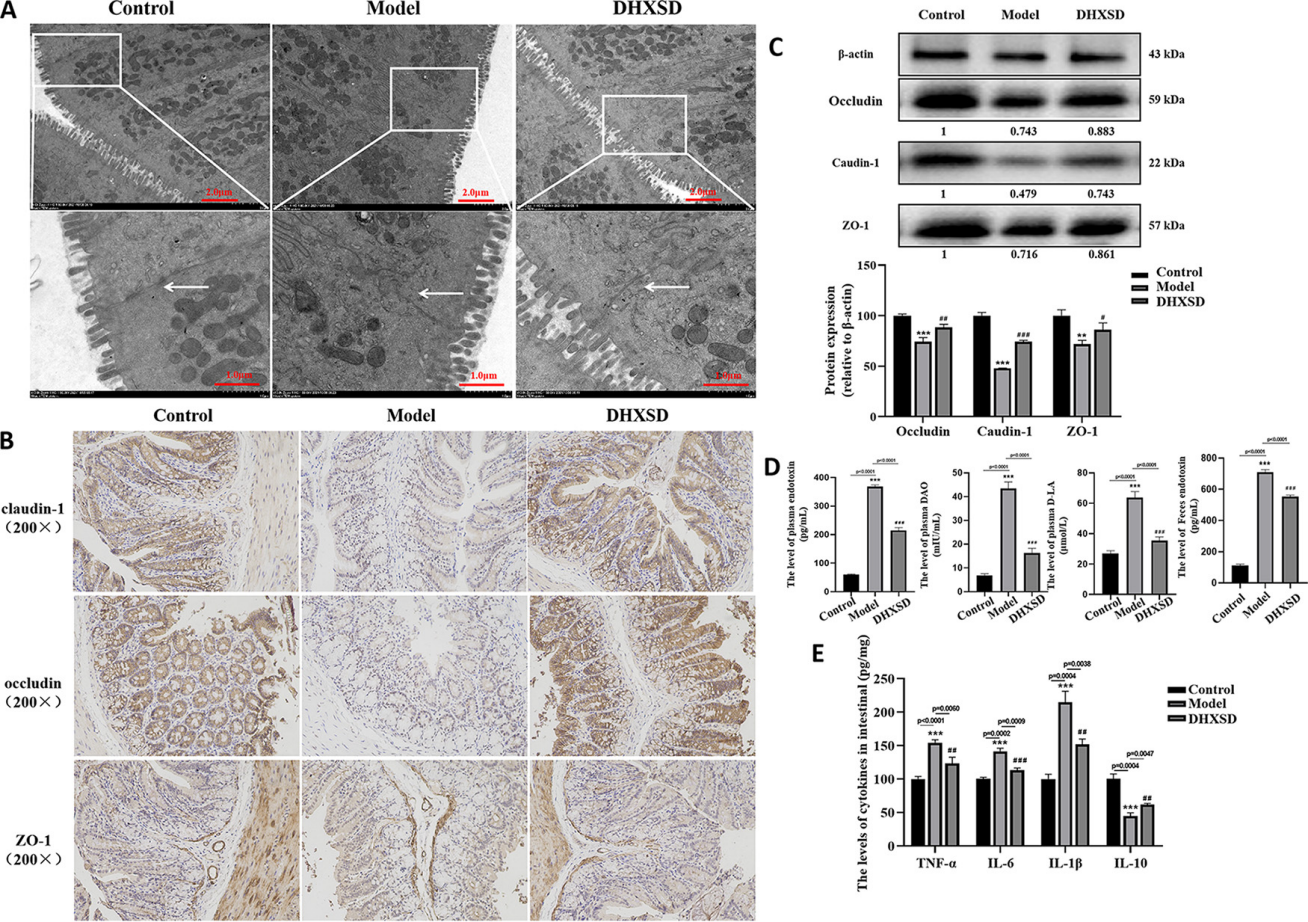
(A) Tight junction structural morphology in ileal mucosa. Location and expression of tight junction protein ZO-1, occludin, and claudin-1 in colonic epithelium from different groups based on the immunohistochemistry (B) and Western blotting (C). (D and E) Effects of DHXSD on ANIT-induced cholestasis intestinal permeability and intestinal immune factors. **, *P* < 0.01 compared with the control group; ^#^, *P* < 0.05 and ^##^, *P* < 0.01 compared with the model group.

To further assess the protective effect of ANIT and DHXSD on the integrity of the colonic epithelial layer, scanning electron microscopy was used to observe the colon. The integrity of the colonic epithelial layer was damaged by ANIT induction (Fig. 2A). Simultaneously, the villi and microvilli of the colonic surface epithelial cells were clearly impaired. However, the administration of DHXSD significantly improved the degree of damage.

### DHXSD protected the integrity of colonic tight junctions in ANIT-induced cholestatisis.

The integrity of the intestinal epithelial tight junctions (TJs) seals the gap between adjacent cells, thereby preventing the movement of microbial toxins and other harmful luminal contents across the epithelium (20). TJs are important indicators of the integrity and central physiological properties of intestinal epithelia. The protein levels of claudin-1, occludin, and ZO-1 were examined using immunohistochemistry and Western blot analyses. Immunohistochemistry (Fig. 2B) and Western blotting analyses (Fig. 2C) showed that ANIT reduced the expression of claudin-1, occludin, and ZO-1 compared to in the control group, while DHXSD treatment remarkably reversed this trend.

### DHXSD ameliorated ANIT-induced cholestatic intestinal permeability and decreased intestinal immune factor levels.

The gut barrier is regarded as the first line of defense in the intestinal tract. Lipopolysaccharide (LPS), d-lactate, and plasma diamine oxidase (DAO) levels were measured to assess the permeability and integrity of the gut barrier. As shown in Fig. 2D, the serum levels of LPS, d-lactate, and DAO were obviously increased in the model group compared with the control group, while DHXSD treatment remarkably reversed this trend. Notably, the intestinal levels of LPS, d-lactate, and DAO were significantly decreased in the model group compared with the control group, while DHXSD treatment remarkably reversed this trend. To assess intestinal immune factors (Fig. 2E), IL-1β, IL-6, TNF-α, and IL-10 were measured to illustrate the potential mechanism of intestinal barrier dysfunction in cholestasis and DHXSD. It was demonstrated that ANIT-induced cholestasis increased the levels of IL-1β, IL-6, and TNF-α and decreased the levels of IL-10. DHXSD treatment significantly reversed this trend. The results indicated that cholestasis disrupted the integrity and intestinal immune factors of the gut barrier, while DHXSD improved it.

### Effects of DHXSD on SCFAs.

A recent research report (21, 22) demonstrated that short-chain fatty acids (SCFAs), as ligands of G-protein coupled-receptors (GPCRs), are key regulators of gut microorganisms and host metabolism. Simultaneously, SCFAs not only exert anti-inflammatory effects in the colonic epithelium, but also enhance the intestinal barrier by facilitating tight junction assembly (23, 24). Therefore, SCFAs are key indicators assessment of intestinal homeostasis, especially gut barrier integrity.

**FIG 3:**
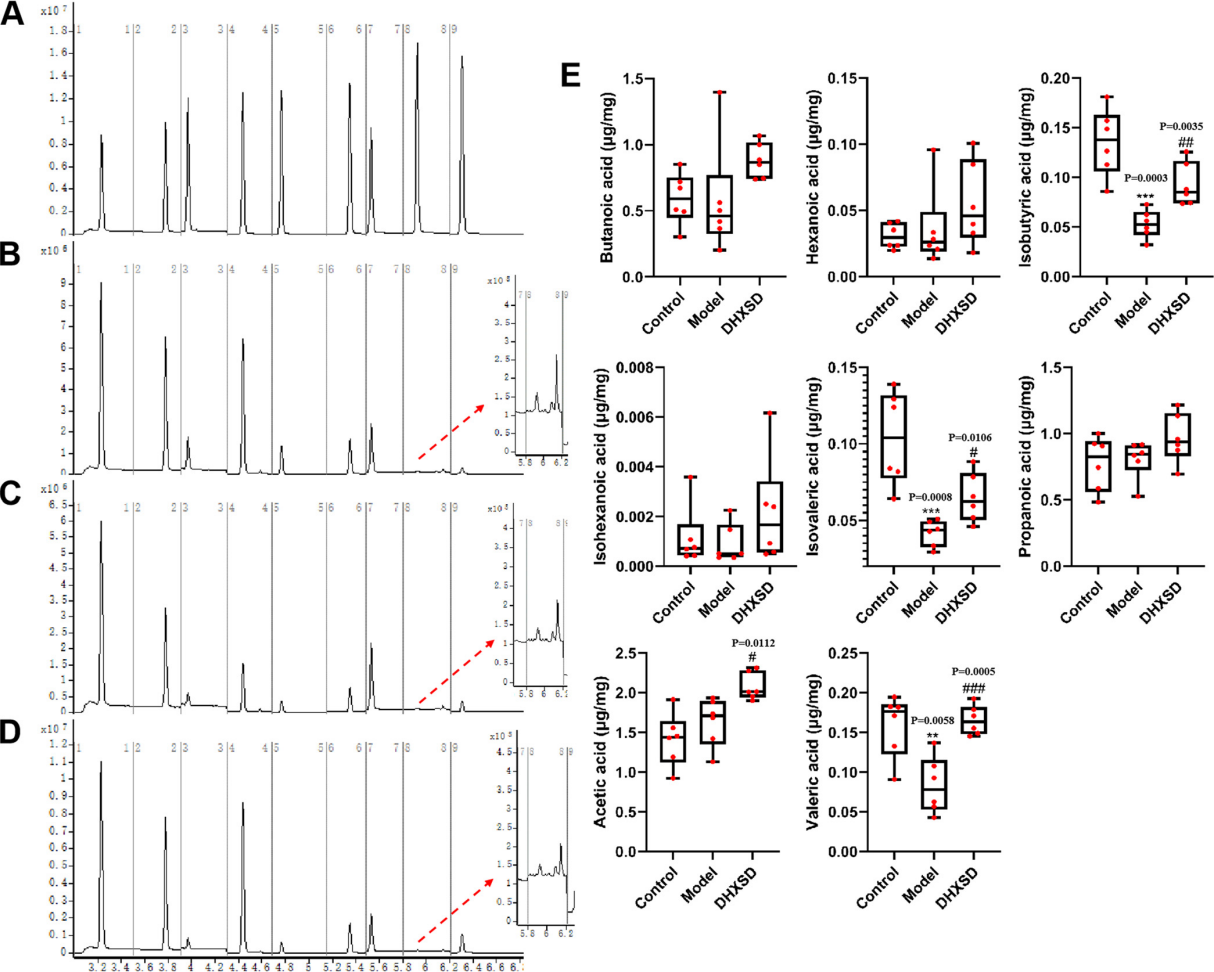
The level of short-chain fatty acids (SCFAs) in the feces from different groups. **, *P* < 0.01 and ***, *P* < 0.001 compared with the control group; ^#^, *P* < 0.05; ^##^, *P* < 0.01; and ^###^, *P* < 0.001 compared with the model group.

In our study, the SCFA content in feces was measured via gas chromatography. The results (Fig. 3) showed that the ANIT group exhibited an obvious decrease in the levels of isobutyric acid, isovaleric acid, and valeric acid compared to the control group. DHXSD supplementation ameliorated ANIT-induced damage to epithelial integrity, accompanied by an increase in isobutyric acid, isovaleric acid, valeric acid, and acetic acid levels.

### DHXSD regulated the gene expression profile.

**FIG 4:**
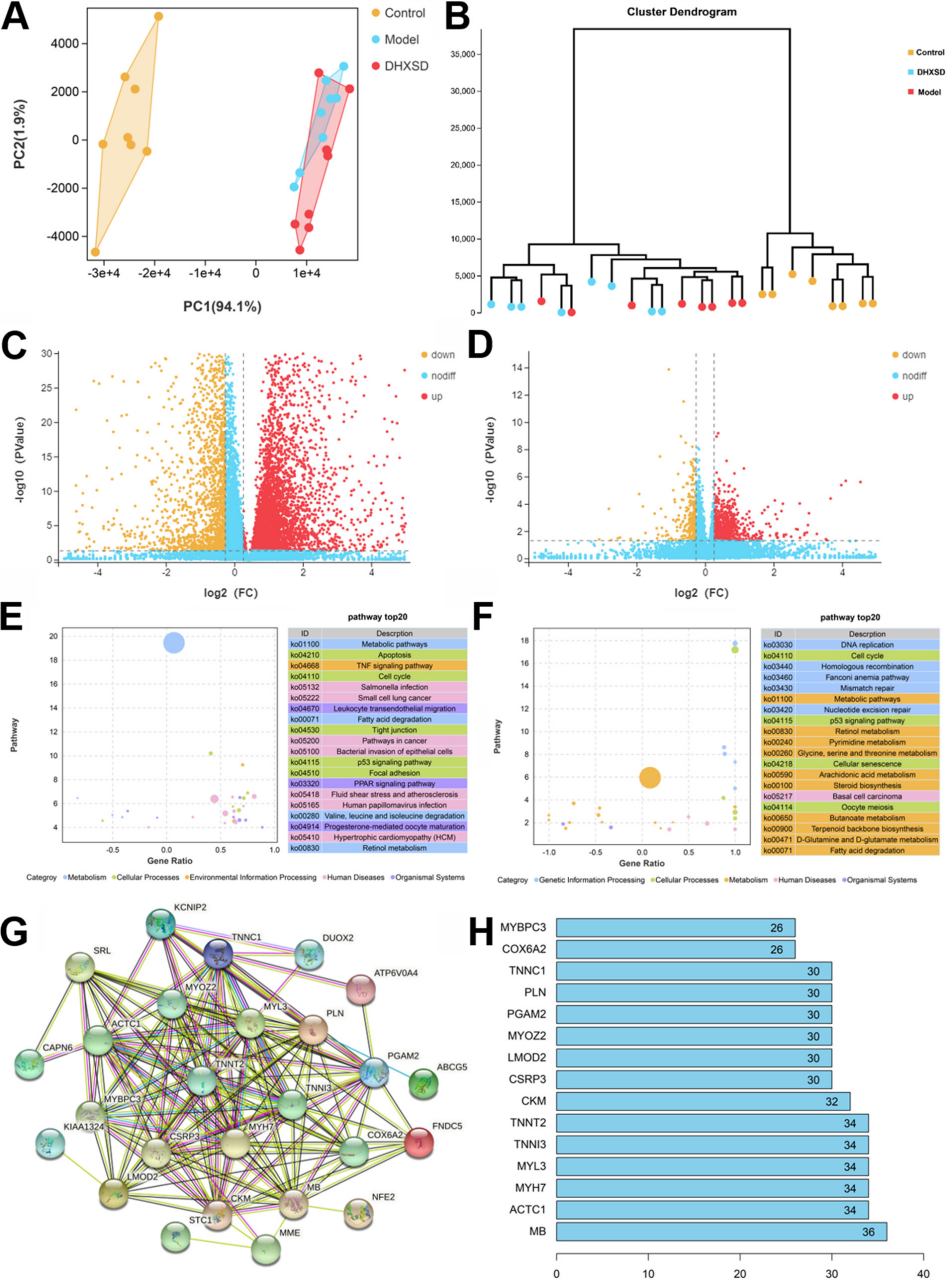
Hepatic transcriptomic analysis of livers from DHXSD-treated mice. (A) Principal coordinate analysis (PCoA) and (B) hierarchical cluster analysis (HCA) was performed to compare the liver RNA-sequencing profiles of different groups. Volcano plots of DEGs in the model versus control group (C) and DHXSD versus model group (D). KEGG enrichment analysis in the gene set 1 (model and control groups; E) and gene set 2 (DHXSD and model groups; F). Protein-protein interaction (PPI) network (G and H) based on the genes which own opposite trends among the control, model, and DHXSD groups.

To demonstrate the target genes of DHXSD for cholestasis, RNAseq analysis was used to obtain the gene expression profile (deposited in the SRA of the NCBI under accession numbers PRJNA893234). According to the results of PCA and the cluster dendrogram (Fig. 4A and B), a clear separation was observed among the three groups, and the model group was separated from both the control and DHXSD groups, demonstrating that the similarity between the control group and the DHXSD group was higher than that of the model group. To better understand the regulation of DHXSD in the gene expression profile, differentially expressed genes (DEGs) were identified with a fold change (FC) of no less than 1.2 and a *P* value ≤ 0.05. A total of 6,671 DEGs between the model and control groups (gene set 1; see Table S1 in the supplemental material) and 1,070 DEGs between the DHXSD and model groups (gene set 2; Table S2) were filtered according to volcano plots (Fig. 4C and D). Among these, 4,663 genes were upregulated, and 2,008 genes were downregulated in the model group. In total, 811 genes were downregulated, and 259 genes were upregulated in the DHXSD group. Interestingly, according to the threshold of FC ≥ 1.2, 2,503 DEGs were obviously upregulated in the model group among gene set 1, and 730 DEGs were remarkably downregulated. Concurrently, 103 DEGs were obviously upregulated after DHXSD treatment, and 26 DEGs were significantly downregulated. Simultaneously, 38 genes (gene set 3; Fig. S1) showed opposite trends in the control, model, and DHXSD groups. A correlation heatmap was applied to represent the covariation between the altered genes and biochemical indices of cholestasis (Fig. S2A). The results showed that SLC34A2, NPAS2, and NFE2 were highly positively correlated with biochemical indices of cholestasis, whereas AABR07044914.1, HPS4, POR, FMO2, ZFP354A, d-glucy, PREX2, USP2, RBM48, PDGFC, ADH6, POLG2, MCM10, LDB1, MCRIP2, TXNIP, URAD, and GPT2 were highly negatively correlated with biochemical indices of cholestasis.

**FIG 5:**
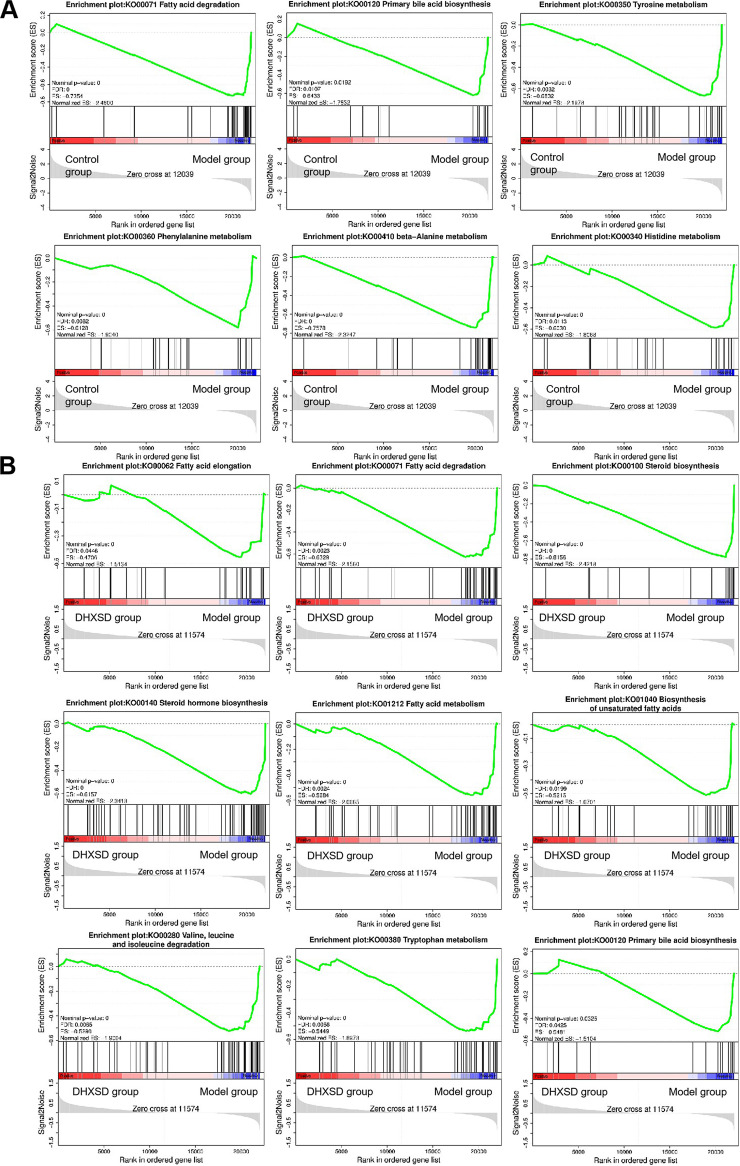
Gene-set enrichment analysis (GSEA) of genes in different groups. (A) Gene sets obtained by GSEA enrichment comparing the model group and the control group. (B) The gene sets obtained by GSEA enrichment comparing the DHXSD group and the model group.

KEGG enrichment analysis was performed on the DEGs. Genes were enriched in pathways related to fatty acid degradation and amino acid metabolism (valine, leucine, and isoleucine degradation and tryptophan metabolism) in gene set 1 (Fig. 4E) and lipid metabolism (arachidonic acid metabolism, steroid biosynthesis, and fatty acid degradation) and amino acids (glycine, serine, and threonine metabolism and d-glutamine and d-glutamate metabolism) in gene set 2 (Fig. 4F). A protein-protein interaction (PPI) network was constructed using the STRING database (https://cn.string-db.org/) based on gene set 3. The results showed that the proteins encoded by these target genes had complex interactions (Fig. 4G and H). Simultaneously, the top 10 nodes (MB, ACTC1, MYH7, MYL3, TNNI3, TNNT2, CKM, CSRP3, LMOD2, and MYOZ2) in the network were considered hub genes (Fig. 2H). Gene set enrichment analysis (GSEA) was performed based on Pearson correlation with phenotypic labels. The GSEA results between the control and model groups illustrated that the model group was highly negatively correlated with the lipid metabolism (fatty acid degradation and steroid hormone biosynthesis), amino acid metabolism (phenylalanine metabolism, beta-alanine metabolism, and tyrosine metabolism), and primary bile acid biosynthesis pathways (Fig. 5A). In addition, the DHXSD group was positively correlated with lipid metabolism (fatty acid elongation, fatty acid degradation, steroid hormone biosynthesis, steroid biosynthesis, fatty acid metabolism, and biosynthesis of unsaturated fatty acids), amino acid metabolism (valine, leucine, isoleucine degradation, and tryptophan metabolism), and primary bile acid biosynthesis pathways (Fig. 5B).

### DHXSD modulated the gut microbiota.

**FIG 6:**
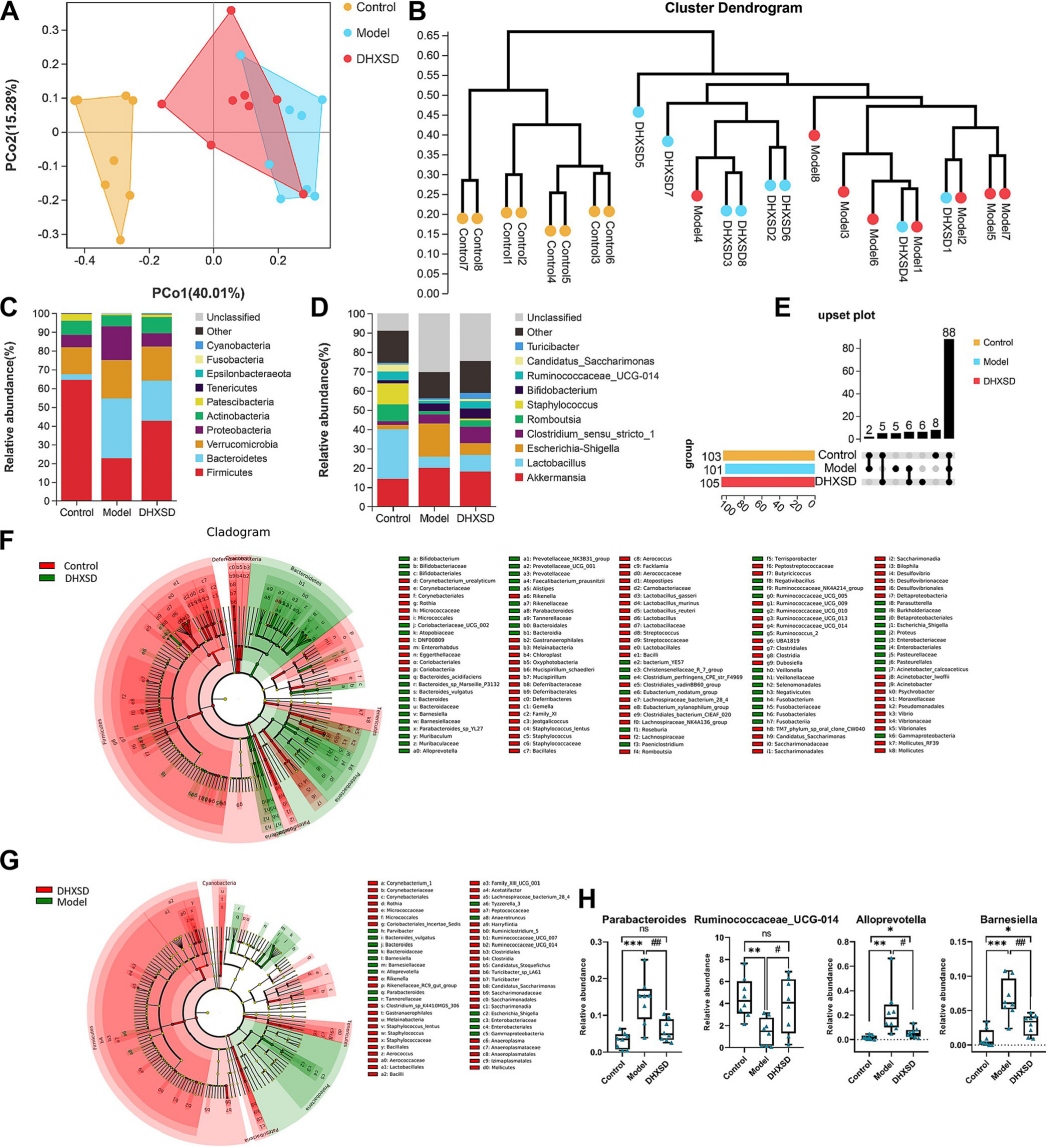
The DHXSD-treated group showed different gut microbiota composition compared with the model group. (A) PCoA and (B) HCA at the OTU level between the three groups. Community distribution at the phylum (C) and genus (D) level. (E) Upset plot based on the gut microbiota among the three groups. LEfSe analysis of the different gut microbiota in the model group compared with control group (F). LEfSe analysis of the different gut microbiota in the DHXSD group compared with model group (G). Potential biomarkers among the control, model, and DHXSD groups (H). *, *P* < 0.05; **, *P* < 0.01; and ***, *P* < 0.001 compared with the control group; ^#^, *P* < 0.05; ^##^, *P* < 0.01; and ^###^, *P* < 0.001 compared with the model group.

16S rRNA sequencing (deposited in the SRA of the NCBI under accession numbers PRJNA893241) was used to investigate the impact of DHXSD on the microbiota. Principal coordinates analysis (PCoA; Fig. 6A) and the unweighted pair group method with arithmetic mean (UPGMA; Fig. 6B) showed that the control, model, and DHXSD groups could be clearly distinguished. Species annotation analysis at the phylum (Fig. 6C, Table S3) and genus levels (Fig. 6D, Table S4) among the three groups illustrated by 16S rRNA sequencing revealed the top 10 abundances. At the phylum level, three of the top 10 most predominant phyla are shown in Fig. 6C. Compared with control group, *Firmicutes* in model group decreased, while *Bacteroidetes* and *Verrucomicrobia* increased. Simultaneously, compared to the model group, *Firmicutes* in the DHXSD group increased; however, *Bacteroidetes* and *Verrucomicrobia* decreased. At the genus level, a noticeable increase of *Akkermansia* and Escherichia-Shigella was observed in model group (Fig. 6D), while *Lactobacillus* decreased compared to control group. Conversely, *Lactobacillus* was markedly upregulated in the DHXSD group compared with the ANIT group, whereas *Akkermansia* and Escherichia*-Shigella* downregulated. An upset plot was constructed to identify shared and host species-specific core operational taxonomic units (OTUs) (Fig. 6E). The results showed that the species-specific core OTUs were relatively low and the shared obviously abundant. Biomarker features in each group were screened using the LEfSe software and Welch's *t* test. Finally, potential biomarkers were screened according to the criteria of “LDA > 2” and “*P* < 0.05” to determine their significance. Finally, 30 (control group versus model group) and 53 (model group versus DHXSD group) significantly altered microbiota at the genus level were screened based on Welch's *t* test analysis and LEfSe analysis (Fig. 6F and G), respectively. Furthermore, 28 microbes at the genus level were screened which were common bacteria often found in the control group versus model group (Table 1) and model group versus DHXSD group (Table 2). Through comparison, a total of four microbial genera (*Parabacteroides*, *Ruminococcaceae_UCG-014*, *Alloprevotella*, *Barnesiella*, Fig. 6H) were simultaneously altered with opposing trends in variation after ANIT and DHXSD treatment. A correlation heatmap was applied to represent the covariation between the altered microbiota and biochemical indices of cholestasis (Fig. S2B). According to the results, *Parabacteroides*, *Alloprevotella*, and *Barnesiella* were positively correlated with biochemical indices of cholestasis, whereas *Ruminococcaceae_UCG-014* was negatively correlated with most of the biochemical indices of cholestasis.

**TABLE 1:** The altered microbes at the genus level between the control group and model group

Phylum	Family	Genus	Control	Model	Fold (model/control)	*P* value	LDA score
*Patescibacteria*	*Saccharimonadaceae*	*Candidatus_Saccharimonas*	3.6794125	0.4181	0.11363227	5.36E-05	4.222608029
*Bacteroidetes*	*Barnesiellaceae*	*Barnesiella*	0.009825	0.067025	6.82188295	0.0003762	3.120425658
*Firmicutes*	*Peptostreptococcaceae*	*Romboutsia*	8.7804875	1.5218125	0.17331754	0.000401418	4.528636633
*Firmicutes*	*Ruminococcaceae*	*Ruminococcaceae_UCG-013*	0.3515125	0.0690875	0.19654351	0.000821468	3.198529192
*Firmicutes*	*Ruminococcaceae*	*Ruminococcaceae_UCG-014*	4.5305375	1.4304375	0.3157324	0.00170147	4.220136458
*Firmicutes*	*Ruminococcaceae*	*Ruminococcaceae_NK4A214_*group	0.058525	0.976025	16.67706109	0.020477677	3.66956206
*Firmicutes*	*Ruminococcaceae*	*Ruminococcaceae_UCG-010*	0.0236875	0.05275	2.22691293	0.039024641	2.738154383
*Bacteroidetes*	*Tannerellaceae*	*Parabacteroides*	0.03165	0.1414375	4.46879937	0.001447485	2.913049611
*Firmicutes*	*Aerococcaceae*	*Aerococcus*	1.9604125	0.01175	0.00599364	0.002945755	3.998128429
*Bacteroidetes*	*Rikenellaceae*	*Alistipes*	0.1229625	0.3939875	3.20412727	0.003315655	3.143096949
*Proteobacteria*	*Desulfovibrionaceae*	*Bilophila*	0.1211	0.0159	0.13129645	0.004388553	2.826103825
*Proteobacteria*	*Desulfovibrionaceae*	*Desulfovibrio*	1.12325	0.1647875	0.14670599	0.014222437	2.826103825
*Firmicutes*	*Family_XIII*	*Eubacterium_nodatum_group*	0.0070625	0.0437375	6.19292035	0.006243899	2.955027815
*Firmicutes*	*Streptococcaceae*	Streptococcus	0.1807875	0.0862	0.47680288	0.006419198	2.879881273
*Firmicutes*	*Staphylococcaceae*	Staphylococcus	10.9750125	0.075925	0.00691799	0.006552467	4.740396654
*Firmicutes*	*Staphylococcaceae*	*Jeotgalicoccus*	0.1058	0.00225	0.02126654	0.022986844	2.872394647
*Firmicutes*	*Lactobacillaceae*	*Lactobacillus*	25.625475	5.60565	0.21875302	0.006660597	4.96799732
*Actinobacteria*	*Eggerthellaceae*	DNF00809	0.1687625	0.0687	0.40708096	0.007585655	2.875267989
*Actinobacteria*	*Eggerthellaceae*	*Enterorhabdus*	1.3461125	0.4687	0.34818784	0.009292297	3.642524764
*Proteobacteria*	*Enterobacteriaceae*	Escherichia *-Shigella*	2.38685	17.2476375	7.22610868	0.01101727	4.891886073
*Bacteroidetes*	*Prevotellaceae*	*Alloprevotella*	0.0165375	0.2267125	13.70899471	0.018849754	3.061465812
*Bacteroidetes*	*Prevotellaceae*	*Prevotellaceae_NK3B31_*group	0.046075	2.6117125	56.68393923	0.032929478	3.985211227
*Firmicutes*	*Lachnospiraceae*	*Eubacterium_xylanophilum_*group	0.044175	0.01575	0.3565365	0.026567083	2.986935586
*Firmicutes*	*Lachnospiraceae*	*Roseburia*	0.008225	0.027075	3.29179331	0.028776452	3.021013788
*Firmicutes*	*Lachnospiraceae*	*Lachnospiraceae_NK4A136_*group	2.81415	0.572525	0.20344509	0.041853476	4.011340212
*Actinobacteria*	*Atopobiaceae*	*Coriobacteriaceae_UCG-002*	0.040975	0.418175	10.20561318	0.035949088	3.280928121
*Actinobacteria*	*Bifidobacteriaceae*	*Bifidobacterium*	1.424325	4.192225	2.94330648	0.0468452	4.159490314
*Proteobacteria*	*Moraxellaceae*	*Psychrobacter*	0.02515	0.004275	0.16998012	0.04909049	2.889410481

**TABLE 2:** The altered microbes at the genus level between model group and DHXSD group

Phylum	Family	Genus	Model	DHXSD	Fold (DHXSD/model)	*P* value	LDA score
*Firmicutes*	*Ruminococcaceae*	*Ruminococcaceae_UCG-014*	1.4304375	3.8250375	2.67403329	0.034353374	4.156410358
*Firmicutes*	*Erysipelotrichaceae*	*Turicibacter*	0.705425	2.982225	4.22755786	0.017210937	4.032151522
*Bacteroidetes*	*Prevotellaceae*	*Alloprevotella*	0.2267125	0.055375	0.24425208	0.043002279	2.983705603
*Bacteroidetes*	*Tannerellaceae*	*Parabacteroides*	0.1414375	0.0583	0.4121962	0.007587197	2.824296653
*Bacteroidetes*	*Bacteroidales*	*Barnesiella*	0.067025	0.0296125	0.44181276	0.006596906	3.241542786
*Actinobacteria*	*Eggerthellaceae*	*Parvibacter*	0.0573625	0.00355	0.06188712	0.024245406	3.08869793

**FIG 7:**
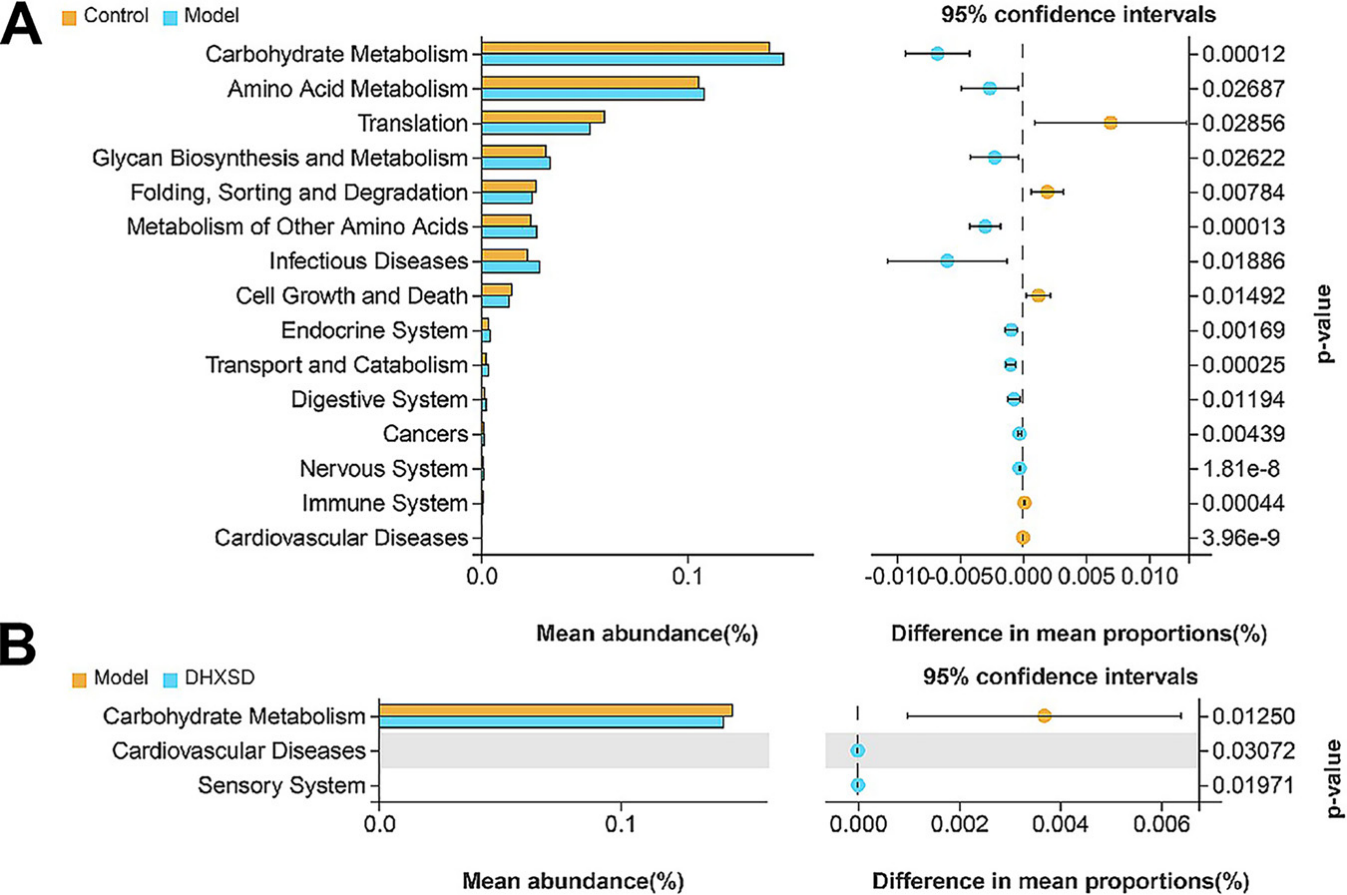
KEGG pathway analysis of the OTUs based on Tax4Fun in the model group compared with control group (A). KEGG pathway analysis of the OTUs based on Tax4Fun in the DHXSD group compared with model group (B).

For function prediction, KEGG pathway analysis of OTUs was performed using Tax4Fun. These differential microbes were mainly associated with lipid metabolism, amino acid metabolism, energy metabolism, and carbohydrate metabolism, etc. (Fig. 7A) in the ANIT group compared with the control group. Fig. 7B showed that the pathways of amino acid metabolism and carbohydrate metabolism, among others, were related to DHXSD treatment compared to the ANIT group.

### Correlation analysis for differential genes and microbes.

**FIG 8:**
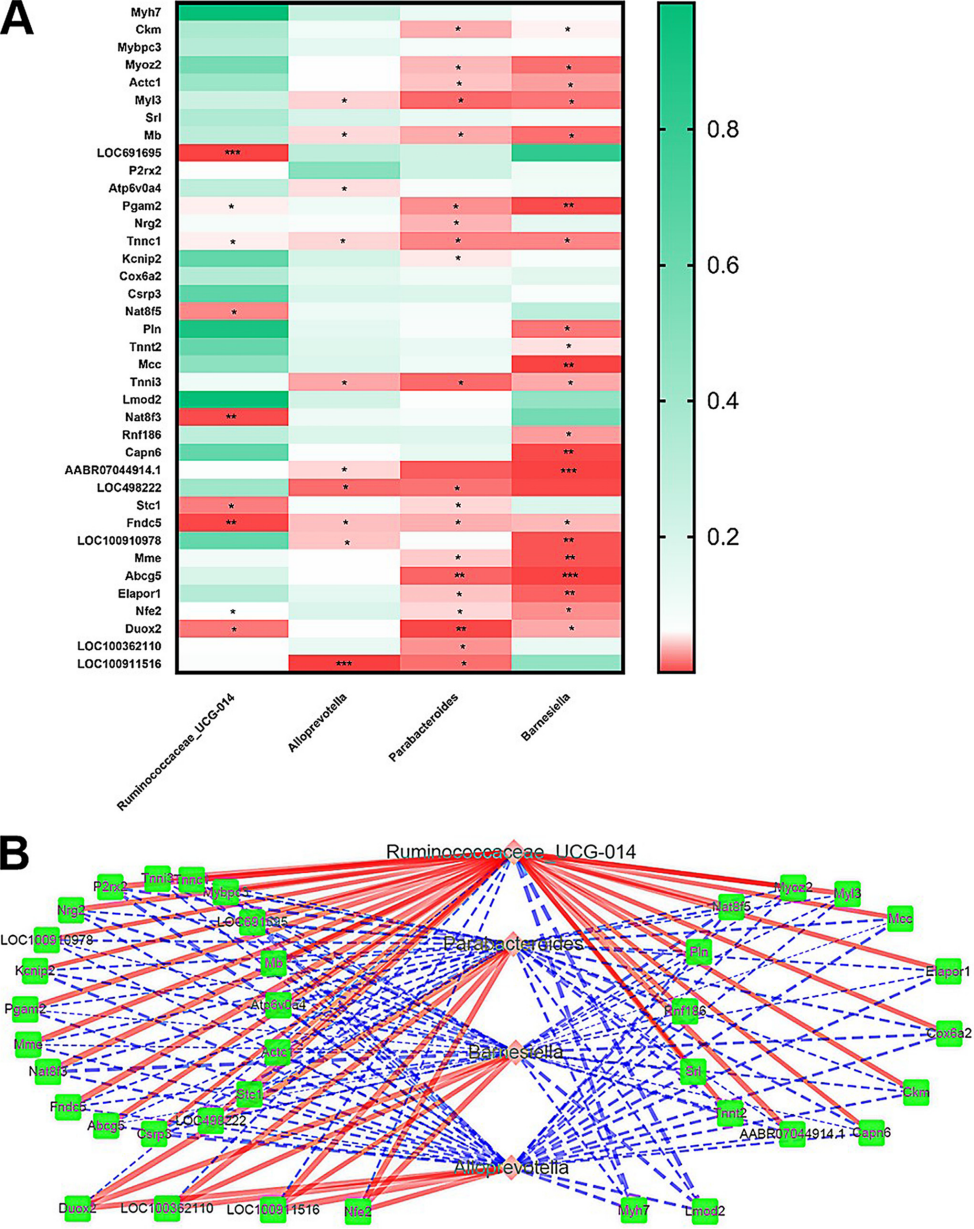
(A) Correlation heatmap is applied to represent the correlation values between perturbed gut microbe genus and altered differentially expressed genes. The asterisk (*) refers to the significance level of the corresponding correlation coefficient in each group. *, *P* < 0.05; **, *P* < 0.01; and ***, *P* < 0.001. (B) Interaction network diagram of the genes-microbes pathways based on correlation analysis. The red and blue lines represent a positive correlation and negative correlation, respectively, between the genes and microbes.

A correlation heatmap and network diagram were constructed to evaluate the covariation between altered gut microbiota genera and altered genes, as shown in Fig. 8A and B. Moreover, the gut microbiota and differential genes were significantly correlated, demonstrating that changes in these genes may be associated with gut microbiota disruption. According to the results, *Alloprevotella*, *Barnesiella* and *Parabacteroides* were negatively correlated with most of the differential genes (except for DUOX2, LOC100362110, LOC100911516, and NFE2), whereas *Ruminococcaceae_UCG-014* was positively correlated with most of the differential genes (except for DUOX2, LOC100362110, LOC100911516, NFE2, MYH7 and LMOD2). Interestingly, *Alloprevotella* was strongly associated with LOC100911516. Simultaneously, *Ruminococcaceae_UCG-014* is related to LOC691695, NAT8F3 and FNDC5. DUOX2 and ABCG5 play prominent roles in cholestasis which is associated with *Barnesiella* and *Parabacteroides*.

According to previous research, the genus *Ruminococcaceae_UCG-*014 belongs to the family *Ruminococcaceae*, the producer of butyrate, and is associated with the progression of the liver disease via increased inflammation and protumorigenic stimulation (25). Simultaneously, *Ruminococcaceae* is known to produce the cholesterol 7α-dehydroxylase (CYP7A1), contributing to the synthesis of bile acid from cholesterol (26). Notably, the genus *Alloprevotella* belongs to the family *Prevotellaceae*, which produces SCFAs, including acetic acid, propionic acid, butyric acid, lactic acid, isobutyric acid, isovaleric acid, and isohexanoic acid (27, 28). SCFAs maintain intestinal epithelium and permeability (29) and regulate intestinal metabolic metabolism via the microbiota-gut-brain axis (30). The genus *Barnesiella* is associated with glutamate/bile acid production and bile salt hydrolase (BSH) activity which deconjugates taurine-conjugated bile acids to form unconjugated bile acids (31). Notably, the genus *Barnesiella* is associated with various immunoregulatory cells and has been suggested to influence the gut environment (32). Within the *Bacteroidetes* phylum, *Parabacteroides* is strongly correlated with the production of anti-inflammatory metabolites (e.g., SCFAs) and the levels of glutamate and gamma-aminobutyric acid (GABA) (33, 34).

## DISCUSSION

Cholestasis is a common liver disease, and new therapeutic options are required. In long-term clinical practice, DHXSD is superior in the treatment of cholestatic liver disease. The aims of this study were to assess the therapeutic effects of DHXSD on cholestasis, and to illustrate the relevant mechanisms based on a multiomics approach (transcriptome and microbiome).

Serum biochemical indices (ALT, AST, ALP, BIL-T, BIL-D, TBA, and GGT) are sensitive indices of liver damage. IL-1β, IL-6, TNF-α, and IL-10 are common indices used to assess the inflammatory response, oxidative stress, and apoptosis, among others, in cholestasis (35, 36). The results of this study illustrated that administration of DHXSD led to a clear improvement in liver damage induced by ANIT. Transcriptome and microbiome analyses in this study also showed that the occurrence of cholestasis is closely related to gut microbiota-regulated amino acid, lipid, and carbohydrate metabolism.

### Lipid metabolism.

Lipid metabolism is a complex biochemical reaction that is necessary for maintaining cell homeostasis, including the digestion, absorption, synthesis, and decomposition of fat under a complex network of enzymes (37). Lipids, ranging from hydrophilic to hydrophobic, play a vital role in the biomolecular membranes that surround all cells and organelles (38). The main functional lipids include structural phosphoglycolipids, sphingolipids, and cholesterol in cellular membranes; triglycerides and free fatty acids as storage lipids; bile acids; and steroid hormones (39). Among cells, the synthesis, absorption, and digestion of different types of lipids are affected by different types of enzymes.

From Fig. S3A, 126 DEGs were found which refer to lipid metabolism in the ANIT-induced model group compared to the control group. Simultaneously, 8 DEGs (COMT, CYP2C22, FADS1, MSMO1, CYP51, DHCR24, CYP2C12, and HMGCS1) were obviously upregulated, while 20 DEGs (CYP2C13, CYP2C11, CYP8B1, CYP4A2, CYP4A1, CYP4A3, CYP23/3A1, CYP2E1, CYP3A18, CYP2D3, CYP2C7, HSD17B2, AKR1C14, ACOX1, ACSL1, HMGCS2, AMACR, ACI1, ADH6, and ALDA3A2) were obviously downregulated in the model group compared with the control group. In contrast, four DEGs (CYP2C7, HMGCS1, CYP1A2, and CYP2C6V1) were upregulated after DHXSD treatment (Fig. S3C). Cytochrome P450 (CYP) monooxygenases were significantly related to cholestasis and DHXSD treatment. According to previous research, CYP enzymes play a key role in the biotransformation of xenobiotics (alcohol, drugs, carcinogens, and environmental pollutants) and endobiotic compounds (steroids, fatty acids, and bile acids) (40). Among the DEGs, CYP8B1 plays a vital role in the ratio of primary bile salts, which might provide clues about the evolution of the bile pathway (41). CYP4A, which consists of 11 subfamilies, is a hydroxylase enzyme that plays a key role in ω-hydroxylation of fatty acids (3). CYP2C enzymes comprise 20% of the total CYP and are present predominantly in the liver; they are important in the biotransformation of bioactive molecules (e.g., biotransformation) (42). 3-hydroxy-3-methylglutaryl-coenzyme A synthase 1 (HMGCS1) plays an important role in cholesterol biosynthesis (43). All DEGs involved metabolic pathways in the control group versus the model group and the model group versus the DHXSD group were shown in Fig. S3B and S3D, respectively.

### Amino acid metabolism.

A variety of amino acids (such as valine, isoleucine, and alanine) can be converted into glucose via gluconeogenesis to replenish energy for the body. The analysis of gene expression in our research revealed 50 DEGs (Fig. S4A), which refer to amino acid metabolism in the ANIT-induced model group, compared to the control group. Simultaneously, eight DEGs (HMGCS1, GSTM3l, ODC1, GSS, SAT1, COMT, GSTT3, and LOC103690139) were obviously upregulated, while 20 DEGs (CAT, CDO1, HADH, ADH6, GPT2, OAT, HAL, ACAT1, CSAD, GNMT, AHCY, INMT, MAT1A, RGD1307603, ACOX1, HMGCS2, ALAS1, SDS, CPS1, and KYNU) were downregulated in the model group compared with the control group. Four DEGs (HMGCS1, LOC103690139, CYP1A2, and BHMT) were downregulated after DHXSD treatment, and ADH6 was obviously upregulated (Fig. S4C). The DEGs associated with amino acid metabolism were responsible for the changes in amino acid content. Ornithine decarboxylase (ODC) is the rate-limiting enzyme in polyamine biosynthesis that catalyzes ornithine metabolism (44). These enzymes of serine acetyltransferase 1 (SAT1) convert l-serine to O-acetyl-l-serine and played a vital role in the synthesis and metabolism of glutamate and serine (45). Interestingly, GSTT3, the largest family of GSTs, is highly expressed in the liver and plays a vital role in hepatotoxicant-induced liver damage, especially individual susceptibility, according to a prebiopsy procedure (46). Simultaneously, GSTT3 correlates well with the transformation of biomarkers in liver injury, such as ALT, BIL-T, and bile acid (47). Cysteine dioxygenase (CDO) is encoded by the CDO1 gene and catalyzes the biotransformation of cysteine to cysteine sulfinate. Cysteine sulfinate can be transformed into pyruvate and sulfite by transamination reactions or hypotaurine and taurine during decarboxylation reactions. The liver-specific gene methionine adenosyltransferase 1A (MATA1), encoding S-adenosylmethionine (SAM) synthesizing isozymes MATI/III, plays an important role in liver damage. SAM can be transformed into S-adenosyl-homocysteine (SAH) via transmethylation reactions or converted to polyamine during decarboxylation reactions (48). All DEGs involved metabolic pathways in the control versus model groups and model versus DHXSD groups are shown in Fig. S4B and S4D, respectively.

### Carbohydrate metabolism.

In the human body, the liver is an essential metabolic organ with various functions, potentially playing a central role in carbohydrate metabolism through carbon metabolism, gluconeogenesis, and fructose and mannose metabolism (49). In addition, the dysregulation of carbohydrate metabolism can result in serious health problems, such as type 2 diabetes, obesity, liver disease, atherosclerosis, and even cancer (50–52). The analysis of gene expression in our study revealed 77 DEGs (Fig. S5A), which refer to carbohydrate metabolism in the ANIT-induced model group, compared to the control group. Interestingly, 11 DEGs (CAT, ADH6, UGP2, SORD, FBP1, ACOX1, RGN, GULO, HAO2, HMGCS2, and PCK1) were significantly downregulated, while HMGCS1 was upregulated in the model group compared to the control group. Similarly, 24 DEGs (Fig. S5C) were screened that refer to carbohydrate metabolism in the DHXSD treatment group compared to the model group. Accordingly, HMGCS1 expression was significantly downregulated following DHXSD treatment. All DEGs involved metabolic pathways in the control versus model groups and model versus DHXSD groups are shown in Fig. S5B and S5D, respectively.

### Studies of the gut microbiota communities.

The LEfSe analysis and Welch's *t* test analysis results showed that 28 differential microbes at the genus level occurred significantly in the control and ANIT-induced model groups, such as *Candidatus_Saccharimonas*, *Barnesiella*, *Romboutsia*, and *Ruminococcaceae_UCG-013*. The genera *Lactobacillus*, *Bifidobacterium*, *Ruminococcaceae*, Escherichia*-Shigella*, Streptococcus, and *Bifidobacterium* are well known alcohol producers (ethanol, propanol, 1,2-propanediol, and 2,3-butanediol production) (53). Simultaneously, these microbes can also produce some important SCFAs, including formate, acetate, lactate, propionate, succinate, and valerate. Gut microbes contribute to increased H_2_S production, especially the bacterial species belonging to the *Desulfovibrionaceae* and *Enterobacteriaceae* families, and the play multiple roles in many physiological processes related to inflammation and immunity (54).

### Conclusions.

In the present study, ANIT-induced liver injury transcriptomic analysis combined with 16S rRNA gene sequencing analysis was utilized to study the mechanisms of the cholestatic effects of DHXSD. Using transcriptomic analysis, 4,663 genes were downregulated, and 2,008 genes were upregulated in the model versus control groups according to volcano plots. In total, 811 genes were downregulated, and 259 genes were upregulated in the DHXSD group. Simultaneously, 68 genes showed opposite trends in the control, model, and DHXSD groups. Concurrently, ANIT-induced liver injury is associated with lipid metabolism (fatty acid degradation and steroid hormone biosynthesis), amino acid metabolism (phenylalanine metabolism, beta-alanine, metabolism, and tyrosine metabolism), and primary bile acid biosynthesis pathways. In addition, the DHXSD group was positively correlated with lipid metabolism (fatty acid elongation, fatty acid degradation, steroid hormone biosynthesis, steroid biosynthesis, fatty acid metabolism, and biosynthesis of unsaturated fatty acids), amino acid metabolism (valine, leucine, isoleucine degradation, and tryptophan metabolism), and primary bile acid biosynthesis pathways. According to 16S rRNA gene sequencing analysis, there are 28 microbes at the genus level were screened which were common bacteria often found in the control and model groups. Through comparison, four microbial genera (*Parabacteroides*, *Ruminococcaceae_UCG-014*, *Alloprevotella*, and *Barnesiella*; Fig. 6H) were simultaneously altered with opposing trends in variation after ANIT and DHXSD treatment. These differential microbes were mainly associated with lipid, amino acid, energy, and carbohydrate metabolism in the ANIT group. The results showed that the pathways of amino acid and carbohydrate metabolism were related to DHXSD treatment. Overall, our research has illustrated that cholestasis is associated with metabolic and microbial dysbiosis and indicates that DHXSD could improve metabolic disorders of cholestasis via the gut microbiota. Simultaneously, this research provides a way to understand the metabolic mechanisms of cholestasis and the use of the gut microbiota as a drug target in treating cholestasis.

## MATERIALS AND METHODS

### Chemicals and reagents.

HB (collection in Sichuan, 20042901), ZZ (collection in Fujian, 210303), DH (collection in Gansu, 20201211), and MX (collection in Jiangsu, 21032501) were obtained from Simcare Ltd. Primary antibodies against ZO-1 (21773-1-AP, 1:1000), occludin (13409-1-AP, 1:1000), claudin-1 (13050-1-AP, 1:1000), and the second antibody of beta-actin (20536-1-AP, 1:10000) were purchased from Proteintech Group Inc. Primary antibodies against NTCP (ab131084, 1:1000), BSEP (ab155421, 1:1000), CYP7A1 (ab234982, 1:1000), MRP2 (ab172630, 1:1000), and FXR1 (ab129089, 1:1000) were obtained from Abcam Inc. ANIT (N106389), UDCA(U110695), and olive oil (O108685) were purchased from the Aladdin Chemical Reagent Co. Dimethyl sulfoxide (DMSO) was purchased from Sigma-Aldrich (reference D4540) (St-Louis, MO, USA). The TRIzol total RNA extraction kit was obtained from Life Technologies.

### Preparation of the DHXSD.

DHXSD was prepared according to a previous study by our research group (55). The weighed powders of DH, HB, and ZZ were immersed and decocted. This procedure was repeated twice, and MX was then added to the decoction. The extract was then decanted and concentrated to 2 g/mL.

### Animals, treatments, and sample preparation.

**(i) Animals and treatments.** Sprague-Dawley rats (*n* = 40) weighing 200 ± 20 g were purchased from Shanghai SIPPR-BK Laboratory Animal Co. Ltd. (permission no. SCXK[Hu] 2018-0006, Shanghai, China). During the experimental period, the room temperature and relative humidity were set to 20 ± 2°C and 50% ± 20%, respectively. The rats were acclimatized to a 12-h light/dark cycle with food and water available *ad libitum*. The experiments were performed in accordance with the Guidelines for the Care and Use of Laboratory Animals and approved by the Animal Ethics Committee of Nanjing University of Chinese Medicine. Rats were randomly divided into four groups of 10 animals each. The rats in the control group were administered normal saline each day and treated with vehicle (olive oil) alone. Rats in the ANIT, DHXSD, and UDCA groups were administered normal saline. On the fifth day, rats were treated with 100 mg/kg ANIT (dissolved in olive oil) to induce cholestasis. Rats in the DHXSD group received a dose of 4.72 g/kg DHXSD for seven consecutive days. Rats in the UDCA group were received a continuous intragastric administration of UDCA (60 mg/kg) for 7 days. All rats were fasted for 12 h and allowed free access to water prior to administration. Blood samples were collected from the abdominal aorta into nonanticoagulant tubes. Blood, liver, intestinal, and stool samples were collected for biochemical, histological, and transcriptional tests, transmission electron microscopy (TEM), and microbiome tests.

**(ii) Blood biochemical profiling assays.** Blood samples were kept at 4°C for 1 h to clot before centrifugation for 10 min at 5,000 rpm at 4°C to obtain serum samples. Serum samples were separated and directly frozen at −80°C until use. The concentrations of alanine transaminase (ALT), alkaline phosphatase (ALP), aspartate transaminase (AST), total bilirubin (BIL-T), direct bilirubin (BIL-D), total bile acid (TBA), and γ-glutamyl transpeptidase (GGT) were measured using an automatic chemistry analyser (Chemray 240; Rayto Life and Analytical Sciences Co., Ltd., China).

**(iii) Histological staining.** For histological analysis, tissues were fixed in 4% paraformaldehyde at 4°C overnight and embedded in paraffin. Formalin-fixed liver sections were cut into 5-μm sections and stained with hematoxylin (H9627, Sigma-Aldrich) and eosin (E4009, Sigma-Aldrich). The results were examined using a Vectra Polaris imaging system (Akoya).

**(iv) Western blotting.** Liver and colon tissues were homogenized using RIPA lysis buffer (Millipore, MA, USA) supplemented with protease phosphatase inhibitor. Protein concentration was detected using the bicinchoninic acid (BCA) method. Equal amounts of protein (50 μg) were loaded onto a sodium dodecyl sulfate-polyacrylamide gel electrophoresis (SDS-PAGE) gel and transferred to polyvinylidene difluoride (PVDF) membranes (previously activated with methanol). The membrane was then blocked with 5% skimmed milk for 1 h and incubated with primary antibodies overnight at 4°C. The PVDF membranes were incubated with secondary antibodies for 1 h at room temperature after three washes with Tris-buffered saline with Tween 20 (TBST). An ECL Plus chemiluminescence reagent kit (Bio-Rad, USA) was used to enhance the protein bands. Reactive bands were quantified using ImageJ software.

**(v) Transmission electron microscopy (TEM).** The intestinal tissues were fixed for 2 h in 4% glutaraldehyde at 4°C and embedded in Epon mixture (Epon 812: acetone = 1:1). Tissues were sliced into 60 to 80-nm sections and stained with uranyl acetate and lead citrate. They were then observed using a Hitachi HT7700 (Hitachi, Tokyo, Japan) transmission electron microscope.

**(vi) Immunohistochemistry.** Tissue sections were deparaffinized and washed with PBS (pH 7.4) for 15 min. The sections were quenched with 3% hydrogen peroxide and blocked with 3% bovine serum albumin (BSA) for 30 min. The sections were incubated with primary antibodies against claudin-1 (1:300, GB11032, Servicebio, Wuhan, China), occludin (1:500, GB111401, Servicebio, Wuhan, China) and occludin (1:500, GB111401, Servicebio, Wuhan, China) at 4°C overnight. The next day, the sections were washed three times with phosphate-buffered saline (PBS, pH 7.4) and incubated with secondary antibodies labeled with horseradish peroxidase (1:200, GB23303, Servicebio, Wuhan, China) at room temperature for 50 min. The slides were placed in PBS, washed three times using a decolourising shaker, and visualized using 3,3′-diaminobenzidine tetrahydrochloride (DAB) condensed chromogen (G1211, Servicebio, Wuhan, China). The slides were counterstained with hematoxylin (G1004, Servicebio, Wuhan, China), and visualized using the Vectra Polaris Imaging System (Akoya, Biosciences, USA).

**(vii) Short-chain fatty acid detection.** A sample of rat feces was collected, and 25 mg of the sample was weighed, and then transferred to 1.5-mL tubes. Water (500 μL, containing 0.5% phosphoric acid) was added to the tubes, grounded (50 Hz) for 3 min, and subjected to ultrasound testing at a low temperature for 10 min. The samples were centrifuged at 13,000 × *g* for 15 min at 4°C to collect the supernatant. The supernatant was transferred into a 1.5-mL centrifuge tube and 0.2 mL N-butyl alcohol solvent (10 μg/mL of internal standard of 2-ethylbutyric acid) was added. The samples were vortexed for 10 s, then centrifuged at 13,000 × *g* for 5 min at 4°C. The sample was filtered through a 0.22-μm filter and injected into a gas chromatograph (8890 B, Agilent Technologies Inc., CA, USA) coupled with a 5977B MSD mass spectrometer and an HP-FFAP capillary column (30 m × 0.25 mm × 0.25 μm film thickness, Agilent J&W Scientific, Folsom, CA, USA). Chromatographic parameters were as follows: flow rate, 1.0 mL/min; injection port, 260°C; injection volume, 1 μL; split ratio, 10:1; solvent delay, 2.5 min. The gas chromatography (GC) oven temperature program applied was as follows: starting temperature of 80°C and ramping at 40°C/min to a temperature of 120°C, then increasing at 10°C/min to 200°C. Ion source temperature and transmission line temperature were all kept at 230°C. The SIM mode for detection and Masshunter software were used for data processing. The absolute content of the SCFA compounds (acetic acid, propanoic acid, butanoic acid, isobutyric acid, valeric acid, isovaleric acid, hexanoic acid, and isohexanoic acid) in the sample was calculated.

**(viii) Enzyme-linked immunosorbent assay and analysis of diamine oxidase.** The concentrations inflammatory cytokines (liver and intestinal samples, IL-1β, TNF-α, IL-6, and IL-10), d-lactate (d-LA), and endotoxin (plasma) were quantified using ELISA kits (Nanjing Jiancheng Bioengineering Institute, Nanjing, China) according to the manufacturer’s instructions (56). After adding the reaction stop solution, the optical density (OD) value was immediately measured using a 450 nm microplate reader, and the results were calculated.

Plasma diamine oxidase (DAO) activity was measured using a DAO assay kit (Nanjing Jiancheng Bioengineering Institute, Nanjing, China), according to the manufacturer’s protocols.

**(ix) Transcriptome analysis.** Total RNA was extracted from rat liver tissues using the TRIzol reagent kit (Invitrogen, Carlsbad, CA, USA) and evaluated using an Agilent 2100 bioanalyzer (Agilent Technologies, Palo Alto, CA, USA). RNA integrity was checked by agarose gel electrophoresis. Eukaryotic mRNAs were enriched using oligonucleotide (dT) beads following RNA extraction. Enriched mRNAs were fragmented in fragmentation buffer and then reverse-transcribed into cDNA using random primers. Fragmentation buffer was used to fragment the enriched mRNA, which was then reverse transcribed into cDNA using the NEBNext Ultra RNA library prep kit for Illumina (New England Biolabs, MA, USA). End repair and poly (A) addition were utilized in the purified double-stranded cDNA fragments, and then ligated to Illumina sequencing adapters. The ligation reaction was purified using AMPure XP beads (1.0 ×), and the size was determined by agarose gel electrophoresis. The ligated fragments were amplified via PCR and sequenced using Illumina NovaSeq 6000.

Adapters or low-quality bases were present in the reads obtained from the sequencing machines, which influenced the assembly and analysis of raw reads. Raw sequencing reads were filtered using FASTP (version 0.18.0), which masked bases with reads containing > 5% N and Q-scores < 20. Clean reads were acquired by removing the rRNA mapped reads using the short reads alignment tool Bowtie2 (version 2.2.8), and then mapping the reference genome using HISAT2. 2.4. StringTie software was utilized to collect the mapped reads based on the reference, and then a fragments per kilobase of transcript per million mapped reads (FPKM) value was established to quantify its expression abundance and variations. DESeq2 software was used to differentially identify mRNAs.

PCA, a statistical procedure, was performed to demonstrate the relationships between data. To further understand the biological functions of genes, the Kyoto Encyclopedia of Genes and Genomes (KEGG) was utilized to filter significantly enriched metabolic pathways in differentially expressed genes (DEGs) compared to the background genome.

** (x) 16S rRNA gene sequencing and bioinformatic analysis.** Fecal bacterial DNA was extracted using HiPure Soil DNA kits (Magen, Guangzhou, China) according to the manufacturer’s protocols. The V3 and V4 regions of the fecal bacterial 16S rRNA genes gene were amplified with primer pairs 341F, CCTACGGGNGGCWGCAG and 806R, GGACTACHVGGGTATCTAAT (57). The resulting PCR products were visualized by agarose gel electrophoresis (2%), further purified using the AxyPrep DNA gel extraction kit (Axygen Biosciences, Union City, CA, USA), and quantified using the ABI StepOnePlus real-time PCR system (Life Technologies, Foster City, CA, USA). To get high quality clean reads, the reads of raw data were removed using FASTP (version 0.18.0) with the following criteria: (i) containing unknown nucleotide “N” over 10%; and (ii) containing less than 50% of bases with a Q value greater than 20. Next, clean reads were merged as raw tags using FLASH (version 1.2.11) and clustered into OTUs ≥97% similarity using the UPARSE (version 9.2.64) pipeline.

Alpha diversity analysis was performed using QIIME. The R ggplot2 package was used to illustrate the OTU rarefaction and rank abundance curves. The phylogenetic tree of beta diversity analysis was performed using FastTree, and a weighted UniFrac distance matrix was generated using the GuniFrac package (version 1.0) in R. Multivariate statistical techniques, including principal coordinates analysis (PCoA) and nonmetric multidimensional scaling (NMDS) of unweighted UniFrac distance, were constructed and plotted using the R Vegan and ggplot2 packages, respectively. For function prediction, KEGG pathway analysis of OTUs was performed using Tax4Fun. Finally, linear discriminant analysis effect size (LEfSe, LDA > 2) and ANOVA were performed to screen species with significant differences at the genus level (*P* < 0.05).

### Data availability.

The data sets used and analyzed during the current study are available from the authors upon reasonable request; some have already been included in this article. Raw sequence data of transcriptome and microbiome which support the findings in our study have been deposited in the SRA of the NCBI under accession numbers PRJNA893234 (Fig. 4, Fig. 5, and Fig. 8) and PRJNA893241 (Fig. 6–8 and Table 1–2), respectively.
