# Supplementary material for: Transcriptome and Gut Microbiota Profiling Analysis of ANIT-Induced Cholestasis and the Effects of Da-Huang-Xiao-Shi Decoction Intervention
Source: Microbiol Spectr. 2022 Nov 21;10(6):e03242-22. doi: 10.1128/spectrum.03242-22 (PMC9769994; doi:10.1128/spectrum.03242-22)
Supplement: Supplemental file 1 — Fig. S1 to S5. Download spectrum.03242-22-s0001.pdf, PDF file, 1.2 MB [file spectrum.03242-22-s0001.pdf]

Supplementary materials

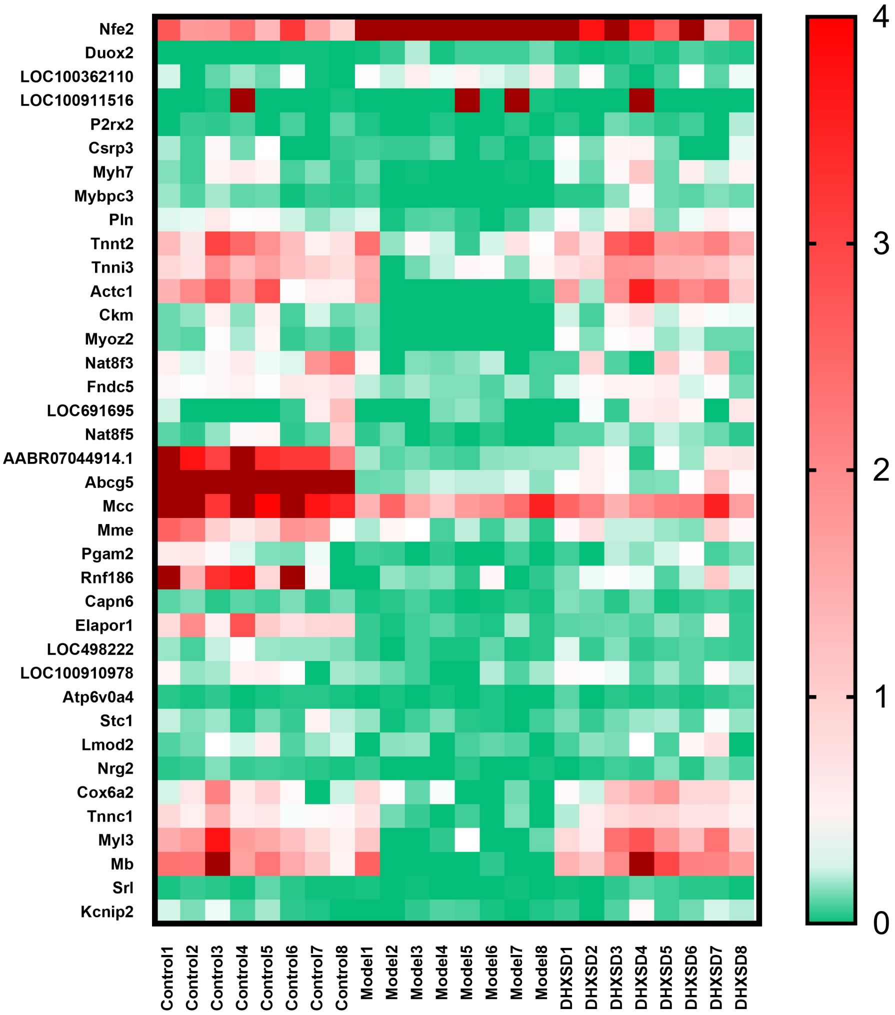

Figure S1 The heat maps of differentially expressed genes among the control, model and DHXSD groups.

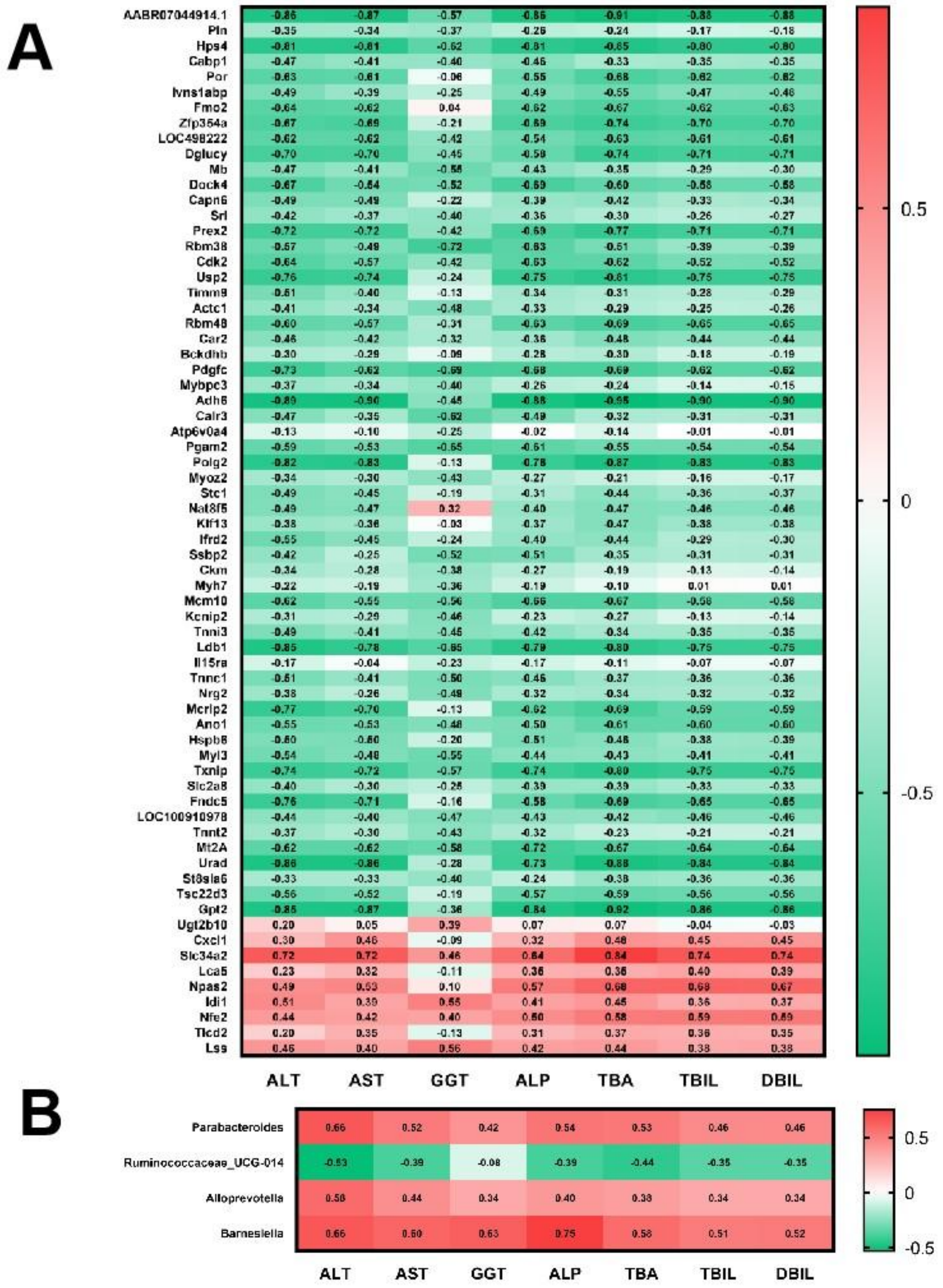

Figure S2 Correlation heatmap was applied to represent the covariation between the altered genes(A)/microbes(B) and biochemical indices of cholestasis.

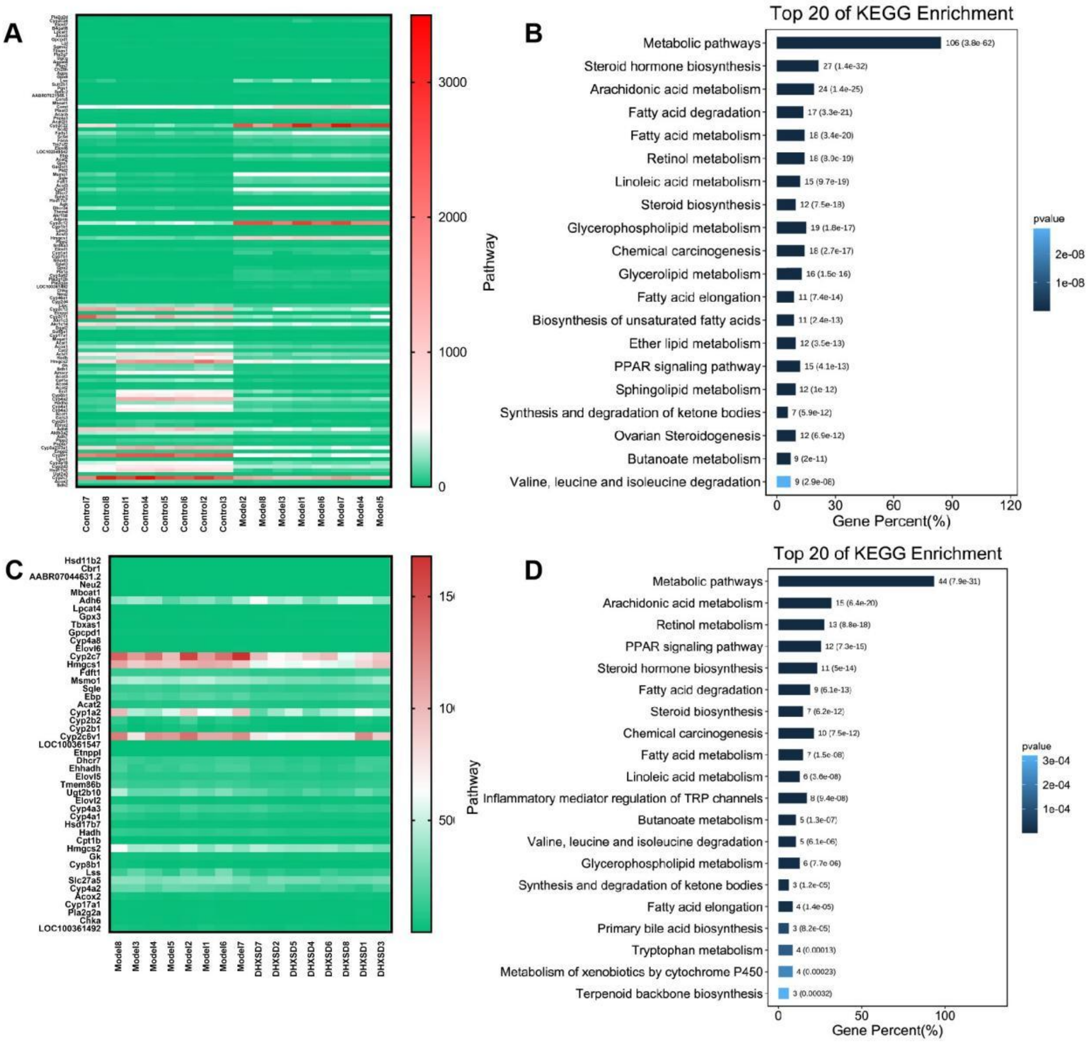

Figure S3 The heat maps of differentially expressed genes (A) and KEGG pathway analysis (B) in the lipid metabolism between model group and control group. The heat maps of differentially expressed genes (C) and KEGG pathway analysis (D) in the lipid metabolism between model group and DHXSD group.

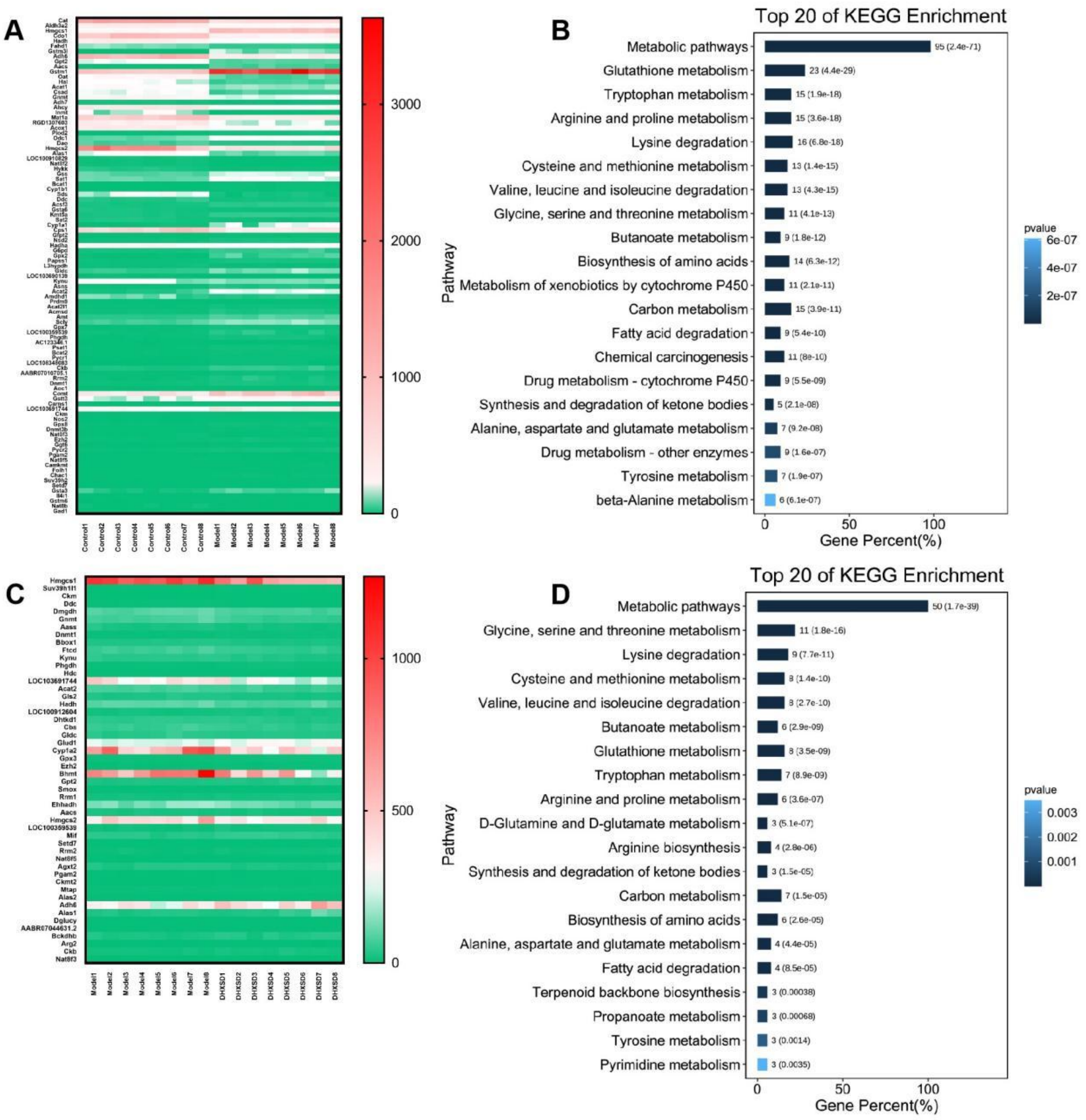

Figure S4 The heat maps of differentially expressed genes (A) and KEGG pathway analysis (B) in the amino acids metabolism between model group and control group. The heat maps of differentially expressed genes (C) and KEGG pathway analysis (D) in the amino acids metabolism between model group and DHXSD group.

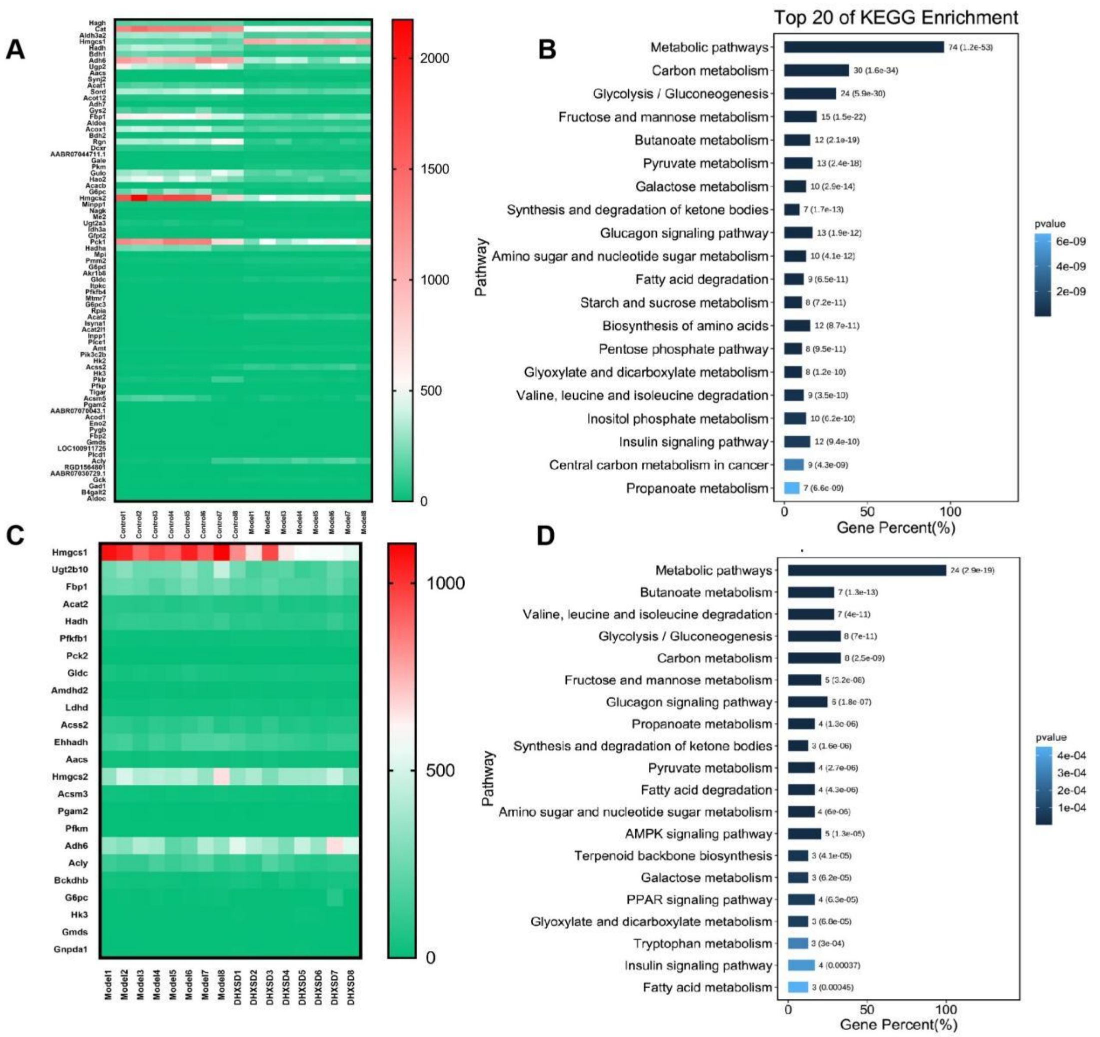

Figure S5 The heat maps of differentially expressed genes (A) and KEGG pathway analysis (B) in the carbohydrate metabolism between model group and control group. The heat maps of differentially expressed genes (C) and KEGG pathway analysis (D) in the carbohydrate metabolism between model group and DHXSD group.
